# Knowledge on postnatal care among postpartum mothers during discharge in maternity hospitals in Asmara: a cross-sectional study

**DOI:** 10.1186/s12884-019-2694-8

**Published:** 2020-01-06

**Authors:** Ghirmay Ghebreigziabher Beraki, Eyasu H. Tesfamariam, Amanuel Gebremichael, Berhanemeskel Yohannes, Kessete Haile, Shewit Tewelde, Simret Goitom

**Affiliations:** 1 Department of Nursing, Orotta College of Medicine and Health Sciences, Asmara, Eritrea; 2 Department of Statistics, Biostatistics and Epidemiology Unit, College of Science, Eritrea Institute of Technology, Mai-Nefhi, Eritrea

**Keywords:** Knowledge, Postpartum mothers, Postnatal care

## Abstract

**Background:**

The early postnatal period is a dangerous time for both mother and baby where morbidity and mortality are highly prevalent if proper care is not done. Post natal care (PNC) knowledge has significant role in reducing such complications. In this study, the knowledge of postpartum mothers on PNC and its determinants were determined.

**Methods:**

A cross-sectional quantitative study was conducted in postpartum mothers (PpM) who attended all maternal delivery services in Asmara. Data was collected by a structured questionnaire. All (*n* = 250) PpM who gave birth in December, 2017 were included in the study. Independent samples t-test and one way ANOVA were used to compare the scores in knowledge across categories of background characteristics using SPSS. Bonferroni post-hoc test was performed for variables that were found to be significant while using ANOVA tool. *P*-values less than 0.05 were considered as significant.

**Results:**

The percentage of PpM who cited vaginal bleeding, as a maternal danger sign, and fever, as a baby danger sign, were 83.2 and 58.8%, respectively. The majority (96%) of PpM responded the correct answer on where to go if they note any danger signs. In addition, more than nine tenth of PpM correctly identified injectable contraceptives (92.7%) and oral contraceptive (91.5%). The percentages of knowledge in recognizing the necessary nutrients ranged from 87.6% for carbohydrates to 46% for minerals. The percentages of correct knowledge regarding first baby bath, frequency of breast feeding, umbilical care, duration of exclusive breast feeding, need and purpose of vaccine were 40.1, 81.9, 77.4, 94.8, and 99.2% respectively. The mean PNC knowledge score was 24.89/60. The score of knowledge on postnatal care was found to significantly differ across the categories of residence (*p* < 0.001) and ethnicity (*p* = 0.015). An increasing trend of knowledge score was observed with increase in age group (*p* < 0.001), educational level (*p* = 0.021), gravida (*p* < 0.001) and para (*p* < 0.001).

**Conclusion:**

Considerable gaps in knowledge regarding postnatal care among postpartum mothers were evident. Special attention should be laid on rural residents, single/living together, junior/below in educational level, primigravida/para, non-Tigrigna ethnicity, and 17 to 25 years old mothers.

## Background

Post-natal care refers to issues pertaining to the mother and the baby from birth up to 6 weeks [1]. The goal of care during the early postnatal period is to promote the physical well-being of both mother and baby, as well as support the developing relationship between the baby and his or her parents and family. In addition, it can also support the development of infant feeding skills and strengthen the mother’s knowledge and confidence in her and her baby’s health and well-being. Accordingly, postnatal care knowledge enables mothers to develop parenting skills to fulfill their mothering role within their particular family [2].

Lack of appropriate postnatal care sometimes may result in death or disability of the mother and/or newborn [3]. Worldwide, nearly 600,000 mothers between the ages of 15–49 years die every year due to complications arising from pregnancy and childbirth. Hence, maternal death occurs almost every minute of every year, out of which 99% are in the developing countries [3]. Around two thirds of maternal and newborn deaths occur in the early postpartum period in developing countries and most of them in sub-Saharan Africa [1, 4]. Almost half of postnatal maternal deaths occur within the first 24 h and 66% occur during the first week [1, 5]. In 2013, 2.8 million newborns died in their first month of life, from which 1 million died on the first day [5].

There has been great emphasis on skilled attendant delivery and efforts have been made to improve PNC guidelines globally and nationally [1]. In sub-Saharan Africa, 48% of women give birth with the assistance of skilled personnel [5]. A review of sub-Saharan Africa demographic and health survey showed that only 13% of women who delivered at home received postnatal care within 2 days of birth [5]. The majority of health care providers across sub-Saharan Africa, including Eritrea, continue to advise mothers to come back to the facility for a first check-up after 6 weeks [6]. Despite these services and advice, maternal and neonatal mortality and morbidity in Eritrea were extremely high [7] . According to a WHO report, infant mortality rate was 36 deaths per 1000 live births and maternal mortality ratio was 501 per 100,000 live births in 2015 [7, 8]. In Eritrea, only 34% of the mothers who give birth were served by trained health workers [9]. Consequently, only 2% of women who had home deliveries receive postnatal care during the first 2 days of post-partum, and another 5 and 7% of such women had postnatal care within 5 to 41 days post-partum [10]. Hence, providing the needed services and advice might not always necessarily lead to achievement of the required goals.

Maternal and child health is one of the basic needs of a society for it is the cornerstone on which a health community and nation are built. Hence, the ministry of health of Eritrea has postnatal care program with sequence of activities that begins with clean delivery practice, followed by clean umblical cord care, thermal care, special care of low birth weight or preterm birth, early and exclusive breastfeeding, as well as immunization programs. However, postnatal care health education given to postpartum mothers in the maternity health facilities of Eritrea is not based on standard guidelines. On the other hand, while discharging the postpartum mothers from the hospital, list of common postnatal danger signs are given in a piece of paper to inform them that they have to approach a nearby health facility upon their appearance.

Postpartum mothers can pass the critical postpartum period successfully if they have knowledge regarding postnatal care [11]. A study conducted in Malawi on assessment of the knowledge and practice of postpartum mothers regarding postnatal care showed that almost all the participants were knowledgeable about some aspect of postnatal care [12]. However, other studies have shown women’s insufficient knowledge on postnatal care [13, 14]. As far as the researchers’ knowledge is concerned, no published resource regarding maternal level of knowledge on postnatal care upon discharge in Eritrea exists. Therefore, this study is designed to determine maternal knowledge regarding PNC and to find out the socio-demographic determinants of knowledge scores on postnatal care among postpartum mothers.

## Methods

### Study design and period

Cross-sectional study design with quantitative approach was used to determine the knowledge of postnatal care among postpartum mothers during discharge in maternity hospitals in Asmara. The study was conducted in December, 2017.

### Study area and population

The study was conducted in the health facilities which provide delivery service in Asmara. Asmara is the capital city of Eritrea, a country in the horn of Africa. This capital city is located 2325 m above sea level with a total area of 44.97km^2^. According to 2017 Asmara municipality report, it has a population size of 416,367. Maternity hospitals in the city are Orotta National Referral Maternity Hospital, Sembel Hospital, Edaga Hamus Community Hospital, and Betmekae Community Hospital. Postpartum mothers who delivered in these four hospitals during the study period constituted the study population.

### Participants

Complete enumeration of the postpartum mothers was undertaken to determine the knowledge of postpartum mothers on postnatal care. This is because all subjects during the specified period of time can be recruited resulting to more accuracy, than that of samples. Hence, all health facilities (4 Hospitals) that render maternity services as well as all eligible postpartum mothers (*N* = 334) who have given birth during the study period in the study area were considered but only 250 were finally included in the study.

### Variables

The dependent variable in the study was the knowledge of postnatal care among mothers who had given birth. The selected determinants were age, marital status, religion, educational level, and occupation.

### Data collection tool and variable measurement

A questionnaire was developed with reference to a guideline prepared by WHO on post-natal care of the mother and new born [1] and previous similar studies conducted in Kenya [15] and Tanzania [16]. After compiling the questionnaire, content validity was assessed using panel of experts from Ministry of Health and Asmara College of Health Sciences. On the other hand, the internal consistency of the tool was computed and found to be within the acceptable range (Richard’s Kurdson = 0.75). Then, the questionnaire was translated from English to Tigrigna, a language most familiar to Eritreans, by experienced researchers, linguists, and midwife experts.

The questionnaire was pre-tested among 30 postpartum mothers in Orotta National Referral Maternity Hospital 1 month before the study period. The interview was done face- to- face by five degree midwife nurses who can speak and understand the language. Pre-designed questions that were not easily understood by the interviewee were simplified after pre-testing the questionnaire. Furthermore, re-arrangement of the questions were made.

The modified questionnaire had two main parts, namely, socio-demographic characteristics and questions that assess knowledge on postnatal care. There were in total 17 questions (with 60 items each having one score) that were used to determine the knowledge on postnatal care encompassing two main components: maternal care, and baby care. Maternal care component consisted of concerns on maternal danger signs (15 items), infection prevention (9 items), bladder care (1 item), sexual activity starting time (1 item), proper nutrition (6 items), delay of menstruation by exclusive breast feeding (1 item), and contraceptive methods (4 items). On the other hand, the baby care component consisted of mechanism of keeping the baby warm (2 items), time of first new born baby bath (1 item), umbilical care (1 item), initiation of breast feeding (1 item), frequency of breast feeding per day (1 item), exclusive breast feeding (1 item), needs and purposes of vaccination (2 items), and baby danger signs (14 items). Every item was scored by assigning one of the following options: “correct” (score = 1), and “wrong” (score = 0). An overall score was obtained by adding the correct responses totaling to 60. The scores indicate that with an increase in score, there is an increase of knowledge regarding postnatal care.

### Data entry and analysis

After verification of the collected questionnaires by the researchers, the data was entered into CSPro (Census and Survey processing system) version 7.0 software package. The entered data was then exported to Statistical Package for Social Sciences (SPSS, version 22.0) for analysis. Frequency (percentage), mean (SD), or median (IQR) were used to describe the data, as appropriate. Normality of the knowledge score was assessed using Kolmogorov-Smirnov test. Independent samples t-test (variables with two categories) and one way ANOVA (variables with more than 2 categories) were used to find out the difference in the level of knowledge of post-natal across demographic variables. Bonferroni post hoc test was performed for the significant ANOVA results. *P*-values less than 0.05 were considered as significant.

### Operational definition

Post-natal care refers to issues pertaining to the mother and the baby from birth up to 6 weeks [1].

Postpartum mothers are those mothers who have given birth in the health facility.

## Results

**Fig. 1:**
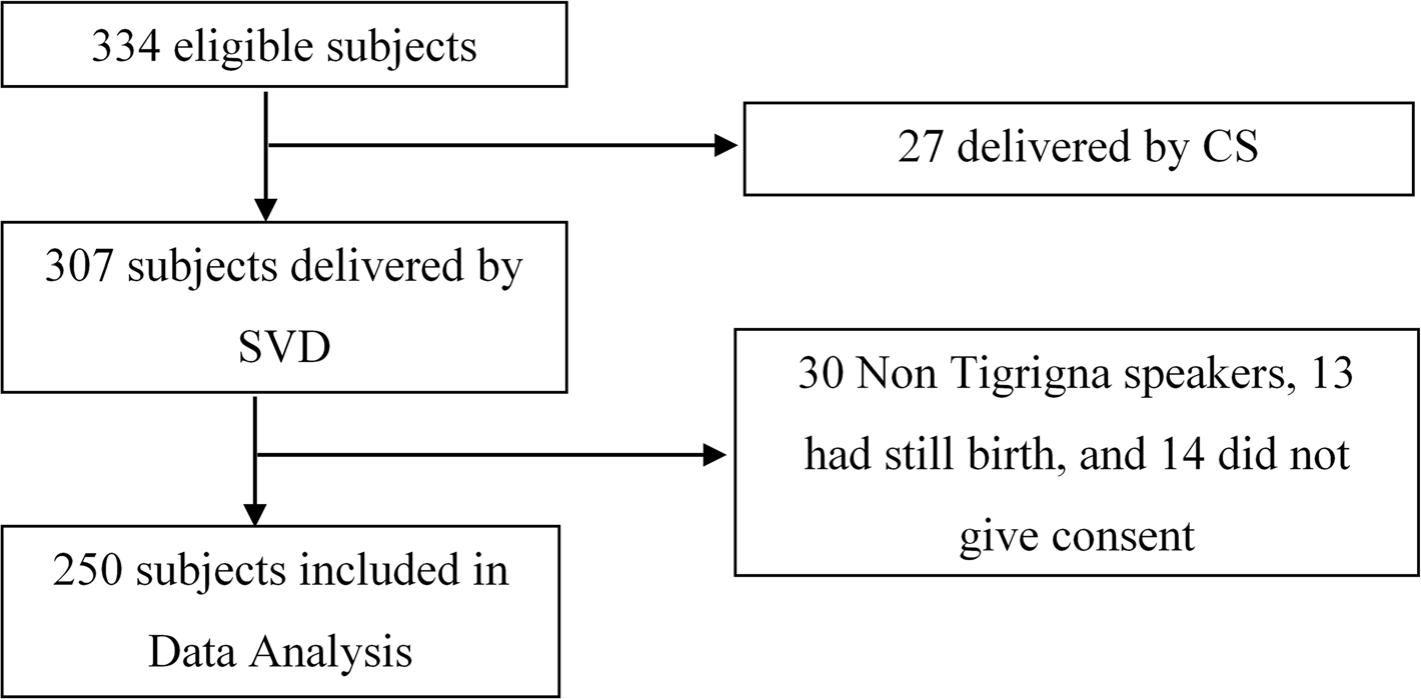
Study participants that were eligible and finally included in the analyses

Data collectors were able to approach 334 postpartum mothers in the four hospitals during the study period. However, 27 delivered by caesarean section and 307 by spontaneous vaginal delivery (SVD). In addition, 30 subjects were excluded because they cannot speak Tigrigna (native language), 13 had still birth, and 14 withdrew from the study to arrive at 250 subjects included in the analysis (Fig. 1).

### Background characteristics of the postpartum mothers

An overview of the socio- demographic characteristics of 250 postpartum mothers during the study period on postnatal care is shown in Table 1.

**Table 1:** Percentage distribution of postpartum mother by demographic characteristics (*n* = 250)

Variables	Frequency	Percentage
Age (Years)		
17 to 25	93	37.2
26 to 30	84	33.6
31 to 42	73	29.2
Residence		
Urban	183	73.2
Rural	67	26.8
Marital status		
Married	232	92.8
Living together	5	2.0
Divorced	2	0.8
Single	11	4.4
Educational level		
Junior and below	73	29.2
Secondary	143	57.2
Higher level	34	13.6
Occupation (*n* = 244)		
Professionals	47	19.3
House wife related	197	80.7
Religion		
Christian	219	87.6
Muslim	31	12.4
Ethnicity		
Tigrigna	234	93.6
Tigre/Saho/Afar	16	6.4

Obstetrical and gynecological history of the mothers revealed that, 28.4, 23.2, 22.8, 8.4, and 17.2% were gravida one, two, three, four, and five and above respectively (Table 2). On the other hand, 33.2% of respondents were primi para, 24.4% were para two, 18.8% were para three, 8.4% were para four and 15.2% were para five and above. Only 37(14.8%) of respondents had history of abortion.

**Table 2:** Percentage distribution of postpartum mothers by obstetrical and gynecological history (*n* = 250)

Variables	Frequency	Percentage
Gravida		
One	71	28.4
Two	58	23.2
Three	57	22.8
Four	21	8.4
Five or above	43	17.2
Para		
One	83	33.2
Two	61	24.4
Three	47	18.8
Four	21	8.4
Five or above	38	15.2
Abortion		
None	213	85.2
One or more	37	14.8

### Knowledge on maternal care

**Fig. 2:**
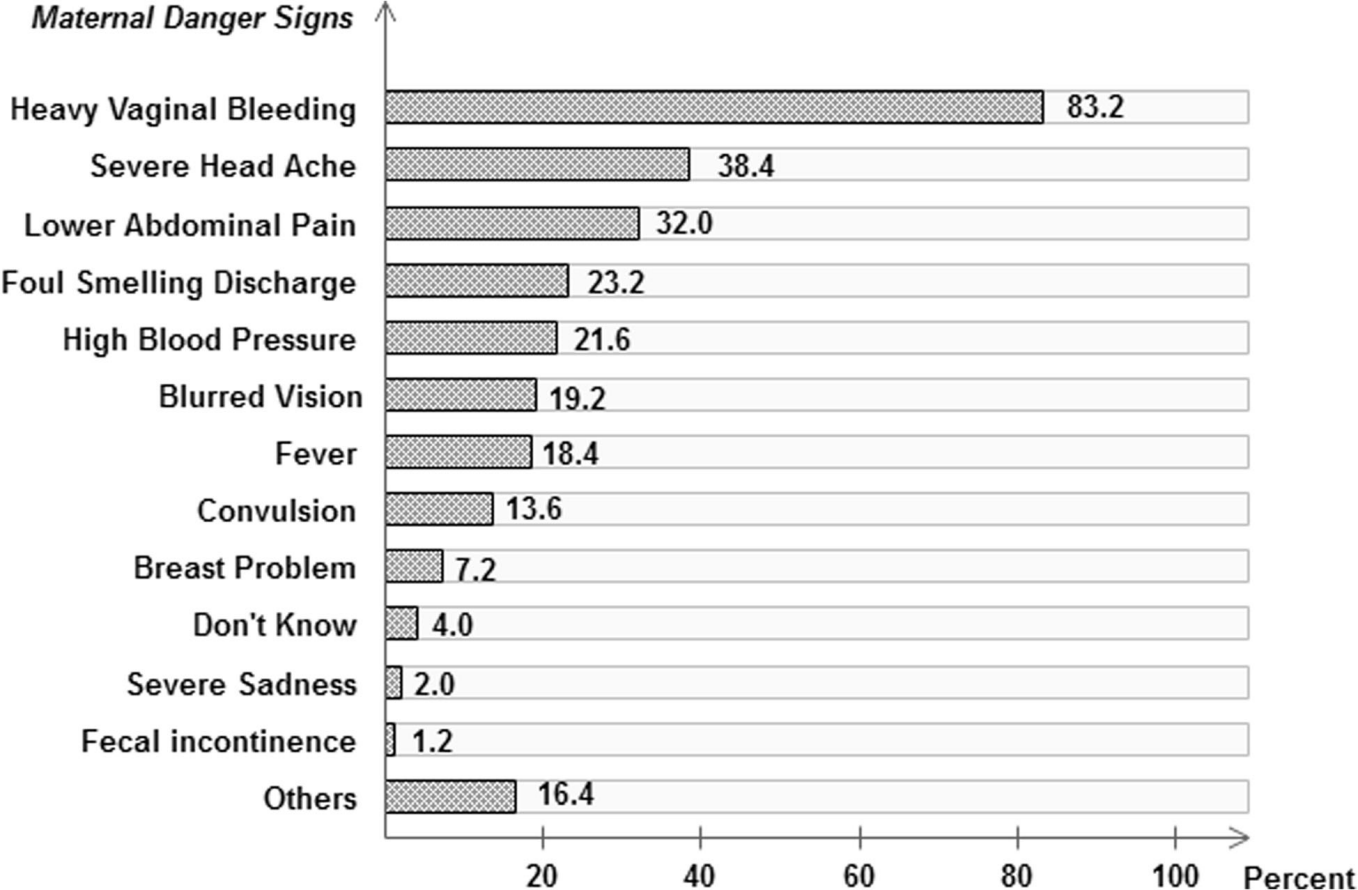
Knowledge of postnatal mothers on maternal danger signs. Others = vomiting, unconsciousness or edema

The percentage distribution of postpartum mothers on maternal danger signs are shown in Fig. 2. The three most recognized maternal danger signs were heavy vaginal bleeding (83.2%), severe head ache (38.4%), and lower abdominal pain (32.0%).

Almost all (96.0%) of the respondents responded correctly on where to go if they note any danger signs (Table 3). Emptying the bladder every 2 hours, which is the correct response for frequency of urination, was mentioned only by 35 (21.6%) of the postpartum mothers. The majority (74.1%) of the respondents mentioned “if I felt to urinate”. The minimum time for starting sexual intercourse was correctly responded by 114 (45.6%). Six different nutrients which are needed to be taken during postpartum were presented to the postpartum mothers for identification. More than 80 % of the postpartum mothers were able to identify food items rich in carbohydrates (87.6%). Moreover, 81.6% replied high fluid intake. Food rich in proteins (75.2%), vitamins (67.6%), fats (47.2%), and minerals (45.6%) were also mentioned. The percentages of women who responded delay in menstrual period as a result of giving exclusive breast feeding for 3 months, 6 months, 1 year, 2 years, and more than 2 years were 12.0, 26.4, 22.8, 17.6 and 21.2 respectively. More than nine tenth of postpartum mothers correctly identified injectable contraceptives (92.7%) and oral contraceptive (91.5%). The remaining had mentioned IUD (53.6%), LAM (16.9%) and other contraceptives (46.8%) such as condom, Norplant or calendar method.

**Table 3:** Percentage distribution of knowledge of mothers on maternal care attributes (n = 250)

Maternal care attributes	Frequency	Percentage
Where to go after danger signs		
Health facility	240	96.0
Stay home or going to holy water	10	4.0
Frequency of urination		
Empty every 2 h	35	21.6
I felt to urinate	120	74.1
I don’t know	7	4.3
Return to sexual activity		
After 42 days	114	45.6
After 3 months	74	29.6
After 6 months	30	12.0
After 1 year	15	6.0
I don’t know	17	6.8
Nutrients		
Carbohydrate	219	87.6
Fluids	204	81.6
Proteins	188	75.2
Vitamins	169	68.0
Fats	118	47.2
Minerals	114	46.0
Delaying of period by exclusive breast feeding		
3 Months	30	12.0
6 Months	66	26.4
1 Year	57	22.8
2 Years	44	17.6
More than two years	53	21.1
Contraceptive method		
Injectable	230	92.7
Oral contraceptive pills	227	91.5
Intrauterine device	133	53.6
Lactational amenorrhea	42	16.9
Condom, Norplant or Calendar method	116	46.8

Nine different infection prevention methods were presented to the postnatal mothers for identification (Table 4). Wash perineum with warm water and some salt (70.0%) and general body hygiene (69.6%) were highly known infection prevention methods. The remaining seven infection prevention methods were known by less than half of the postpartum mothers. Among the least known infection prevention methods were hand washing after changing pads (10.8%), hand washing after perineal hygiene (10.4%), and hand washing before perineal hygiene (6.8%).

**Table 4:** Percentage of postpartum mothers on responses regarding infection prevention methods

Variables	Frequency	Percentage
Hand washing before perineal hygiene	17	6.8
Hand washing after perineal hygiene	26	10.4
Hand washing after changing pads	27	10.8
Changing pads frequently	91	36.4
Changing pants frequently	72	28.8
General body hygiene	174	69.6
Wash with warm water and some salt	175	70.0
Washing after defecation	89	35.6
Washing after urination	82	32.8

### Knowledge on baby care

Table 5 shows the percentage distribution of mothers by their knowledge on baby care. Knowledge on keeping the baby warm by wrapping the baby with cloth was almost universal (99.6%). Few (6.8%) also mentioned skin to skin contact. Correct response on the time at which first bath can be given for a new born baby was obtained from 67(26.8%) of the study participants. More than three fourths (77.4%) of the women responded umbilical care should be simply keeping clean and dry. Most of the respondents (88.40%) mentioned the correct answer on initiation of breast feeding after delivery which is within 30 min. Almost three fourths (74.0%) of the women correctly responded to the frequency need of breast feeding per day (eight per day); however, 10.4% said ‘if the baby cries’. The majority (94.8%) of respondents correctly answered that duration of exclusive breast feeding needs to be for 6 months. Almost all respondents (99.2%) correctly knew the needs of vaccine for a newborn baby. With regards to the purpose of vaccine, most of the respondents (94.8%) mentioned ‘to prevent disease’ and 13 (5.2%) did not know.

**Table 5:** Percentage distribution of knowledge of mothers regarding maternal care attributes (*n* = 250)

Baby care attributes	Frequency	Percentage
Keeping the baby warm^a^		
Skin to skin contact	17	6.8
Wrap the baby with clothe	249	99.6
First baby bath time		
Immediately	35	14.0
After 6 h	27	10.8
After 24 h	67	26.8
After 1 week	17	6.8
After 3 days	83	33.2
I don’t know	21	8.4
Umbilical care mechanism		
Clean and dry	147	77.4
Apply butter	28	14.7
Apply cow dung	1	0.5
Apply vaseline/oil	79	31.7
I don’t know	14	7.4
Initiation of breast feeding		
Within 30 min	221	88.4
Within 1hour	23	9.2
Within two to 3 hours	5	2.0
Within 6 hours	1	0.4
Frequency of breast feeding/day		
At least 8 times	185	74.0
If baby cries	26	10.4
At least 4 to 6 times	24	9.6
I don’t know	15	6.0
Duration of exclusive breast feeding		
3 Months	7	2.8
4 Months	3	1.2
6 Months	237	94.8
1 year	3	1.2
Purpose of vaccination		
To prevent disease	237	94.8
I don’t know	13	5.2

^a^Percent might exceed 100 because of multiple response.

More than half of the respondents mentioned fever (58.8%), severe vomiting (53.2%), and difficulty in breathing (50.8%) as baby danger signs (Table 6). Almost one third of the women were able to identify the inability to breast feed (32.8%) and irritability (33.2%). Less than one fourth of the respondents cited umbilical problems (14.0%), abdominal distention (9.2%), convulsion (8.4%), lethargy (7.6%), yellowness of eyes (7.6%), eye problem (6.8%), yellowness of palms (2.8%), and yellowness of sole (1.6%). However, 19 (7.6%) of the respondents mentioned “I don’t know” and 101 (40.4%) responded vomiting/unconsciousness/ edema.

**Table 6:** Percentage distribution of knowledge of PN mothers on baby danger signs

Variables	Frequency	Percentage
Yellowness of eyes	19	7.6
Yellowness of palms	7	2.8
Yellowness of soles	4	1.6
Umbilical problems	35	14.0
Eye problems	17	6.8
Unable to breast feed	82	32.8
Difficulty in breathing	127	50.8
Convulsion	21	8.4
Fever	147	58.8
Lethargic	19	7.6
Irritable	83	33.2
Abdominal distention	23	9.2
Sever vomiting	133	53.2
I do not know	19	7.6
Vomiting/unconsciousness/edema	101	40.4

### Comparison of knowledge scores

The results showed that the mean knowledge score was 24.89/60 (SD = 5.66). Independent sample t-test has revealed that the categories in residence (*p* < 0.001) and ethnicity (*p* = 0.015) had shown significant difference in score of knowledge on postnatal care among postpartum mothers (Table 7). However, occupation (*p* = 0.210), religion (*p* = 0.476), number of abortion (*p* = 0.783) have not shown significant difference in score of knowledge on postnatal care among postpartum mothers.

**Table 7:** Comparison of knowledge of postpartum mothers on post natal care scores using t-test

Variables	M (SD)	Diff.^a^(95 CI)	*p*-value
Residence			
Urban	25.69 (5.79)	2.97 (1.38, 4.57)	< 0.001
Rural	22.72 (5.31)		
Occupation			
Professional	25.98 (5.92)	1.16 (−0.66, 2.98)	0.210
House wife related	24.82 (5.64)		
Religion			
Christian	24.99 (5.80)	0.79 (−1.40, 2.99)	0.476
Muslim	24.19 (5.88)		
Ethnic group			
Tigrigna	25.12 (5.79)	3.62 (0.69, 6.55)	0.015
Tigre/Saho/Afar	21.50 (5.06)		
Number of abortion			
None	24.93 (5.93)	0.29 (−1.76, 2.33)	0.783
One or more	24.65 (5.12)		

^a^*Diff.* Difference in mean

Result from one way ANOVA (Table 8), revealed that there was significant difference in the average score of knowledge regarding post natal care among the postpartum mothers across different age groups (*p* < 0.001), marital status (*p* = 0.045), educational level (*p* = 0.014), gravidity (*p* < 0.001) and parity (*p* < 0.001). An increasing trend of knowledge score was observed with increase in age group (*p* = 0.001), educational level (*p* = 0.021), gravidity (*p* < 0.001) and parity (*p* < 0.001).

**Table 8:** Comparison of knowledge on post natal care scores using ANOVA test

Variable	M (SD)	F-Value	*P*-value	*P*-value trend	Post Hoc Result^a^
Age group					
17–25	22.88 (5.73)	9.68	< 0.001	< 0.001	^b^17–25 < 26–30 = 31–42
26–30	25.82 (5.66)				
31–42	26.38 (5.42)				
Marital status					
Married	25.16 (5.70)	2.72	0.045	–	Married >LT = Single
Living together	19.80 (6.46)				Married = Divorced
Divorced	25.50 (6.36)				LT < Divorced
Single	21.55 (6.40)				Divorced > Single
Educational level					
Junior or below	23.23 (6.10)	4.38	0.014	0.021	JB < Secondary = HL
Secondary	25.65 (5.69)				
Higher level	25.26 (5.01)				
Gravida					
One	22.03 (5.83)	7.42	< 0.001	< 0.001	One < Two, Three, Four, FA
Two	25.47 (4.87)				Two = Three = Four = FA
Three	26.11 (5.89)				
Four	25.05 (6.37)				
Five or above	27.16 (4.91)				
Para					
One	22.25 (5.53)	5.89	< 0.001	< 0.001	One < Two, Three, Four, FA
Two	25.89 (5.29)				Two = Three = Four = FA
Three	25.89 (6.03)				
Four	25.38 (5.80)				
Five or above	27.55 (4.91)				

^a^Bonferroni post hoc test was performed, *LT* Living together, *JB* Junior or below, *HL* Higher level, *FA*: Five or above

^b^17–25 < 26–30 = 31–42: Knowledge score among 17–25 aged mothers is significantly less than those 26–30 and 31–42. Besides, knowledge score among 26–30 aged mothers is not significantly different from 31 to 42

Bonferroni post-hoc comparison showed that the postnatal knowledge score among mothers aged 17–25 was significantly less than 26–30, and 31–42 years old. Moreover, significantly higher knowledge score was observed among married and divorced as compared to single and living together. Postpartum mothers who are junior or below were also found to have significantly lower postnatal knowledge score as compared to mothers who are secondary and higher level. No significant difference in knowledge score was observed among mothers who were gravidity two, three, four, and five or above, however, mothers of gravidity one had significantly less knowledge score than mothers of the aforementioned gravidity. Similar results with that of the gravidity were observed for parity.

## Discussion

There are maternal and child health programs to safeguard maternal and child health in Eritrea. Nonetheless, maternal and neonatal mortality ratio still remain as high as 501/100,000 and 36/1000 live births, respectively, in the country [8]. Studies regarding utilization of maternal health services such as antenatal care and skilled delivery at birth are not infrequent; however, there still exists paucity of studies on knowledge regarding postnatal care in the country. One of the fundamental activities that needs to be instigated for the improvement of maternal and neonatal health is postnatal care because the majority of maternal and newborn death happen during this period [1]. Among others, one dimension of initiating postpartum care constitutes enhancing the knowledge of the mothers in order to enable them to properly handle themselves and the neonates in times of difficulty [17].

In this study, vaginal bleeding (83.2%) was the most frequently mentioned danger sign during the postpartum period. This finding is similar to a research conducted in Ethiopia (89.2%) [18] but lower than another study done in Nepal (98.47%) [3]. When compared with the study conducted in Ethiopia, a similar result observed is foul-smelling vaginal discharge (23.3%) as a danger sign [18]. However, in the Ethiopian study, relatively higher percentages of postnatal mothers identified severe headache (38.4% Vs 23.1%), blurred vision (19.2% Vs 8.9%), convulsion (13.6% Vs 7.9%) and lower abdominal pain (32.0% Vs 2.9%) as danger signs as compared to this study [18]. This could be due to the long standing public health campaigns given in Eritrea that bleeding either during pregnancy or post-partum period puts the mother’s health at danger.

In this study, the most identified infection prevention methods were washing perineum with warm water and some salt (70.0%) and changing pads frequently (36.4%). These findings are lower than those yielded in the study done in Nepal, in which 91.33% of PpM had prior knowledge of washing perineum with warm water and 83.16% changing pads frequently [3]. This discrepancy could be due to differences in the availability of health facilities and maternal training in the two study populations.

Around half (45.6%) of the postpartum mothers were aware about the appropriate time for restarting sexual intercourse in this study. However, it is difficult to say whether the postpartum mothers’ knowledge on this issue has been obtained through proper and methodical mechanisms, such as trainings and educational interventions, or from tradition. However, it is worth remembering that the scientific and traditional reason of commencing sexual intercourse after 6 weeks has the same ground. Lochia stays up to 6 weeks postpartum putting the mother at risk of postpartum infection and the pain as well as discomfort associated with the childbirth. Besides, the majority (87.6%) of participants in this study were Christians, and specifically belonged to the Orthodox Christian religion, where women are traditionally obliged to abstain from sexual intercourse for a minimum of 40 days because they are considered as polluted (tainted) after childbirth.

Worldwide, unwanted pregnancy is a major cause of death in children less than 5 years of age and a death of pregnant women attempting abortion (650 per100,000 pregnant women) each year [11, 19]. Hence, a reliable contraceptive method is needed for mothers to avoid unwanted pregnancy as early as possible because menstruation period usually restarts at 3 to 9 weeks [11]. In the current study, knowledge of PpM on contraceptive methods shows that injectable contraceptives (92.7%) and oral contraceptives (91.5%) were highly mentioned. These findings are higher than the study done in Nepal that indicated injectable or Depo-Provera (78.0%) followed by oral contraceptive pills (74.0%) as the most common methods [20]. Therefore, it seems that maternal training and consultation about contraceptive methods in Eritrea is properly addressed and should be maintained.

In the present study, regarding knowledge of postpartum mothers on keeping baby warm after delivery, almost all participants (99.6%) had a higher knowledge when compared to a study conducted in Nepal which showed that 82% had knowledge on wrapping the baby with warm clothes. However, in this study, lower level of knowledge (6.8%) was scored on keeping baby warm by skin to skin contact than the study done in Nepal (58%) [21]*.*

Almost one-fourth of the PpM knew the correct answer for ‘when to give a bath to a newly born baby’ which is similar to a study conducted in India (30%) [22]. Around three-fourths of the postpartum mothers in the current study responded that umbilical cord is taken care of by simply keeping the umbilicus clean and dry. However, the study in Nepal showed that 59% were knowledgeable about keeping the umbilicus clean and dry [21]. The culture-driven beliefs and practices have also led 14.7% of the postpartum mothers in this study to indicate that butter is to be applied for umbilical care.

Health education provision by health workers at the health facilities and through the mass media could be the possible reason behind the correct knowledge (88.4%) of postpartum mothers regarding initiation of breastfeeding within 30 min. However, in a study done in Nepal, the percentage of mothers who mentioned that breastfeeding needs to be initiated within 1 hour was 48% [21]. Fever, as a new born danger sign, was identified by only 58.8% of the postpartum mothers in this study. This can be said to be relatively lower than the findings in Ethiopia (76.6%) [18] and Kenya (74.9%) [15]. Moreover, the difficulty of breathing as a baby danger sign was mentioned by almost half (50.8%) of the postpartum mothers. The same danger sign, was known by 46.6% of the participants in Kenyan study [15], whereas the current study finding was higher than that of Ethiopian study (19.7%) [14].

Results regarding new-born danger signs on umbilical cord problem (14, 5.4, 35%), convulsion (8.4, 19.3, 15%), and eye problem (6.8, 16.7, 21%) were not similar in this study, Ethiopia, and Nepal respectively [14, 21]. Yellow palm as baby danger sign was the most highly unidentified (97.2%) by the *postpartum* mothers in this study, which is similar to the findings in Ghana (93.6%) [23]. However, inconsistent results on jaundice as baby danger sign were obtained in a study done in Nepal (21%) [21].

Comparison of the knowledge scores by categories of residence has revealed that urban residents had significantly greater knowledge score than the rural residents. The main reason for the difference could be the regularity in health education in urban places. Another possible reason could be the easy access to transport in urban places to go to the place where health education is offered. The lower level of knowledge among mothers who were in the age group 17 to 25, or primipara could be due to practical lessons that multipara mothers gain each time birth occurs.

### Limitations of the study

The use of a cross sectional design in this study did not allow for causal relationships to be established, thus the reasons why postpartum mothers reported certain maternal and baby care more so than others is not known.

## Conclusion

The average knowledge score on PNC of the postpartum mothers was low for it was below half of the overall score. Moreover, significant association between the mothers’ knowledge and their age group, residence, educational background, and parity was observed. Therefore, special attention should be given to mothers living in rural areas, junior or below in educational level, single or living together, primigravida/primipara, ethnic groups other than Tigrigna, and those between 17 to 25 years of age to improve PNC knowledge.

## Data Availability

Data set is available in electronic form which can be accessed upon a reasonable request from the corresponding author.

## Abbreviations

ANOVA: Analysis Of Variance
CS: Cesarean Section
IQR: Interquartile range
PNC: Post Natal Care
PpM: Postpartum Mothers
SD: Standard Deviation
SPSS: Statistical Package for Social Sciences
SVD: Spontaneous vaginal delivery
WHO: World Health Organization
